# Small RNAs in mycobacteria: an unfolding story

**DOI:** 10.3389/fcimb.2014.00096

**Published:** 2014-07-24

**Authors:** Katie Haning, Seung Hee Cho, Lydia M. Contreras

**Affiliations:** ^1^McKetta Department of Chemical Engineering, Cockrell School of Engineering, The University of Texas at AustinAustin, TX, USA; ^2^Department of Molecular Biosciences, Institute for Cellular and Molecular Biology, The University of Texas at AustinAustin, TX, USA

**Keywords:** small RNA, mycobacteria, regulation, pathogenesis, non-coding RNAs, *Mycobacterium tuberculosis*

## Abstract

Mycobacteria represent a class of powerful pathogens, including those causing tuberculosis and leprosy, which continue to be worldwide health challenges. In the last 20 years, an abundance of non-coding, small RNAs (sRNAs) have been discovered in model bacteria and gained significant attention as regulators of cellular responses, including pathogenesis. Naturally, a search in mycobacteria followed, revealing over 200 sRNAs thus far. Characterization of these sRNAs is only beginning, but differential expression under environmental stresses suggests relevance to mycobacterial pathogenesis. This review provides a comprehensive overview of the current knowledge of sRNAs in mycobacteria, including historical perspective and techniques used for identification and characterization.

## Introduction

**Figure 1:**
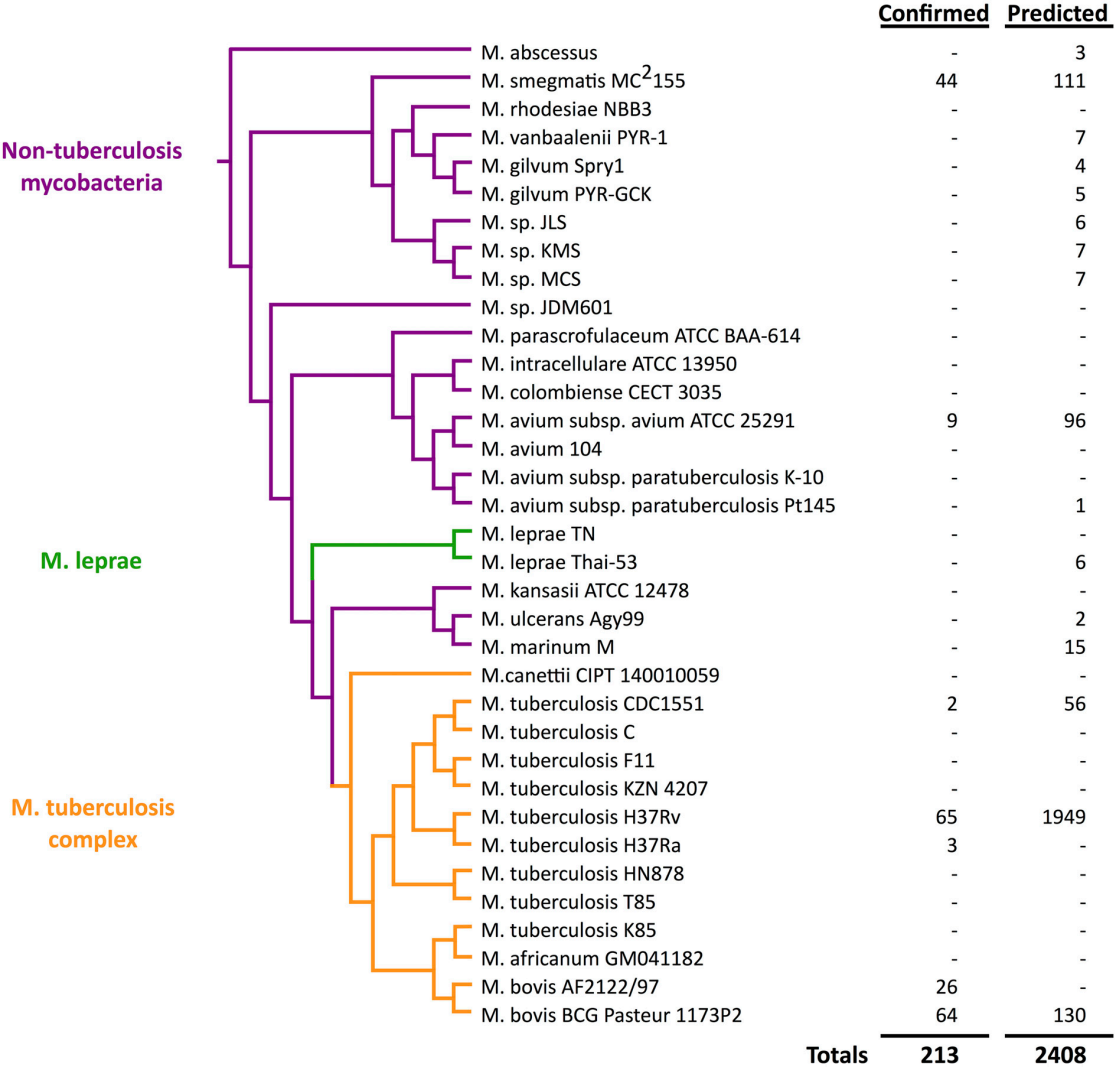
**Quantitative view of sRNA discovery in mycobacteria by phylogeny**. Confirmed sRNAs have been experimentally validated while unconfirmed sRNAs include unverified computational predictions (Wattam et al., 2014).

Mycobacterial species threaten human health worldwide, causing infectious diseases such as tuberculosis and leprosy. More than 140 species have been reported in the gram-positive genus *Mycobacterium*, which is divided into three major categories: *Mycobaterium tuberculosis* complex, *Mycobacterium leprae*, and non-tuberculosis mycobacteria (Jagielski et al., 2014). The majority of species are non-tuberculosis mycobacteria related to non-pathogenic organisms that live in water or soil. Diagnosis of non-tuberculosis mycobacteria is complex since they are diverse in growth temperatures, growth rates, and drug susceptibility, as well as in clinical relevance (Cosma et al., 2003). *M. leprae* and *Mycobacterium ulcerans* are considered to be highly successful pathogens for causing leprosy and Buruli ulcers, respectively. However, *M. tuberculosis* is one of the most common pathogens, causing tuberculosis in humans and animals. *M. bovis* and other five closely related species are also classified within the *M. tuberculosis* complex (shown in Figure 1). Although genome sequences are highly related among species in the *M. tuberculosis* complex, phenotypic properties and hosts vary by species (Cole et al., 1998; Garnier et al., 2003).

*M. tuberculosis* is transmitted by aerosol and has evolutionarily developed various strategies to evade host immune systems. Upon invasion, *M. tuberculosis* infects host macrophages. Infection with attenuated strains of *M. tuberculosis* causes apoptosis to macrophages and, consequently, induces adaptive immunity by recruiting T cells (Behar et al., 2010). However, virulent *M. tuberculosis* might inhibit apoptosis, favoring macrophage necrosis, and ultimately translocate into the cytosol. Once it can survive against macrophages, *M. tuberculosis* starts to quickly replicate its genome and to infect adjacent cells (Keane et al., 2000; Wilkinson et al., 2005). It is worth noting that within granuloma, formed with macrophages, neutrophils, and other immune cells, *M. tuberculosis* persists for a long time through various evasion strategies that can lead to reactivation and, eventually, disease (Huynh et al., 2011).

The life cycle of *M. tuberculosis* is unique in that it has two distinct metabolic states: one is an active replicative state and the other is a persistent state (Wayne and Sohaskey, 2001). *M. tuberculosis* can hide from the host defense mechanism by non-proliferative persistent states. This is the most contributing feature of *M. tuberculosis* for successful survival in host cells and is referred to as latent infection, but can be reactivated. It has been known that continuous activation of macrophage is important for preventing reactivation of the infection (Flynn et al., 1998). As such, control of latency in mycobacteria depends on host responses such as maintenance of granuloma, cytokines, and chemokines (Flynn and Chan, 2001).

Development of effective drugs against mycobacteria is still an active area of research. The keys to these efforts lie in the mechanisms of how mycobacteria switch from latent states to aggressive, disease-causing forms. Recently, sequence-based approaches for mycobacteria revealed extensive numbers of non-coding RNAs that include intergenic small RNAs (sRNAs) (Arnvig et al., 2011). Regulatory RNAs participate in adaptive responses of bacteria against environmental change such as transitions during pathogenesis. As interest escalates in sRNAs across mycobacterial species, there is a need to compile current knowledge in the field to unify and focus future work to gain highly sought medical advantages. The focus of this review is to unfold the history of sRNA investigation in mycobacterial species. We begin with a brief narration of sRNA discovery and characterization in pathogens and then extensively document progress in the search and characterization of mycobacterial sRNAs in particular.

## Small RNAs in pathogenesis: from discovery to targeting

Mycobacteria and other pathogens must be highly adaptive to endure and exploit environmental changes presented by hosts and their immune responses. Traditionally, various transcription factors were credited for such cellular reprogramming by turning on and off expression of relevant genes in response to environmental stimuli. However, over the last 15 years, understanding of bacterial gene expression has expanded beyond the DNA level, now including multiple layers of regulation. Particularly interesting are sRNAs, typically 50–500 nucleotides in length, known to act as global regulators of cellular phenotypes (Gottesman and Storz, 2011). Although previously thought to be strictly untranslated regions, some sRNAs have now been classified as bifunctional, encoding small peptides (Bobrovskyy and Vanderpool, 2013). Although our focus is on sRNAs, we have also included limited discussions of recently identified riboswitches in mycobacteria.

### Efforts of sRNA discovery in model organisms highlight biological relevance

The first bacterial sRNA was discovered almost 50 years ago in *Escherichia coli* MRE600 (ATCC 29417), but its function remained unknown for three decades, surfacing shortly after the complete genome of *E. coli* K-12 was published (Hindley, 1967; Blattner, 1997; Wassarman and Storz, 2000). At this time, 10 sRNAs were known in *E. coli*, mostly discovered by chance during studies of individual genetic systems (Wassarman et al., 1999). However, availability of the fully sequenced *E. coli* K-12 genome led to the booming of computational approaches, enabling large-scale systematic searches of intergenic regions for sRNAs. In fact, 14 novel sRNAs were reported in a single study of *E. coli* in 2001, more than doubling the previously known list (Argaman et al., 2001). Recent developments in deep sequencing and high-density microarray technologies have continued to allow extensive genome-wide studies of sRNAs and their functions. Now, ~100 sRNAs are known to exist in *E. coli* and it has become clear that many are involved in regulating stress responses (Gottesman et al., 2006; Park et al., 2013). These sRNAs show significant conservation among other organisms, including pathogenic bacteria (Hershberg et al., 2003).

Investigations in *E. coli* have increasingly shifted toward mechanistic studies and revealed that regulatory sRNAs typically function by base-pairing with target mRNAs, thereby altering transcription, mRNA stability, or translation (Gottesman, 2004). Regulatory sRNAs are generally classified by their genomic locations with respect to their targets. *Cis*-encoded sRNAs are encoded on the same gene as a target mRNA, but in the opposite, complementary direction, while *trans*-encoded sRNAs are located apart from their targets in the genome. Many *trans*-encoded sRNAs have been shown to depend on Hfq, an RNA-binding protein providing stability, in order to function (Zhang et al., 2003); this has been particularly prevalent in gram-negative bacteria. Some sRNAs have also been discovered to regulate proteins directly (Romeo et al., 2013). Lastly, riboswitches represent another class of powerful RNA regulators, typically found in the 5′ untranslated regions (5′ UTRs) of their mRNA targets and directly responsive to environmental changes. In this category, RNA thermometers have been found to respond structurally to changes in temperature to regulate translation (Kortmann and Narberhaus, 2012). It is worth noting that sRNA classification continues to present a major challenge with increasing diversity.

**Figure 2:**
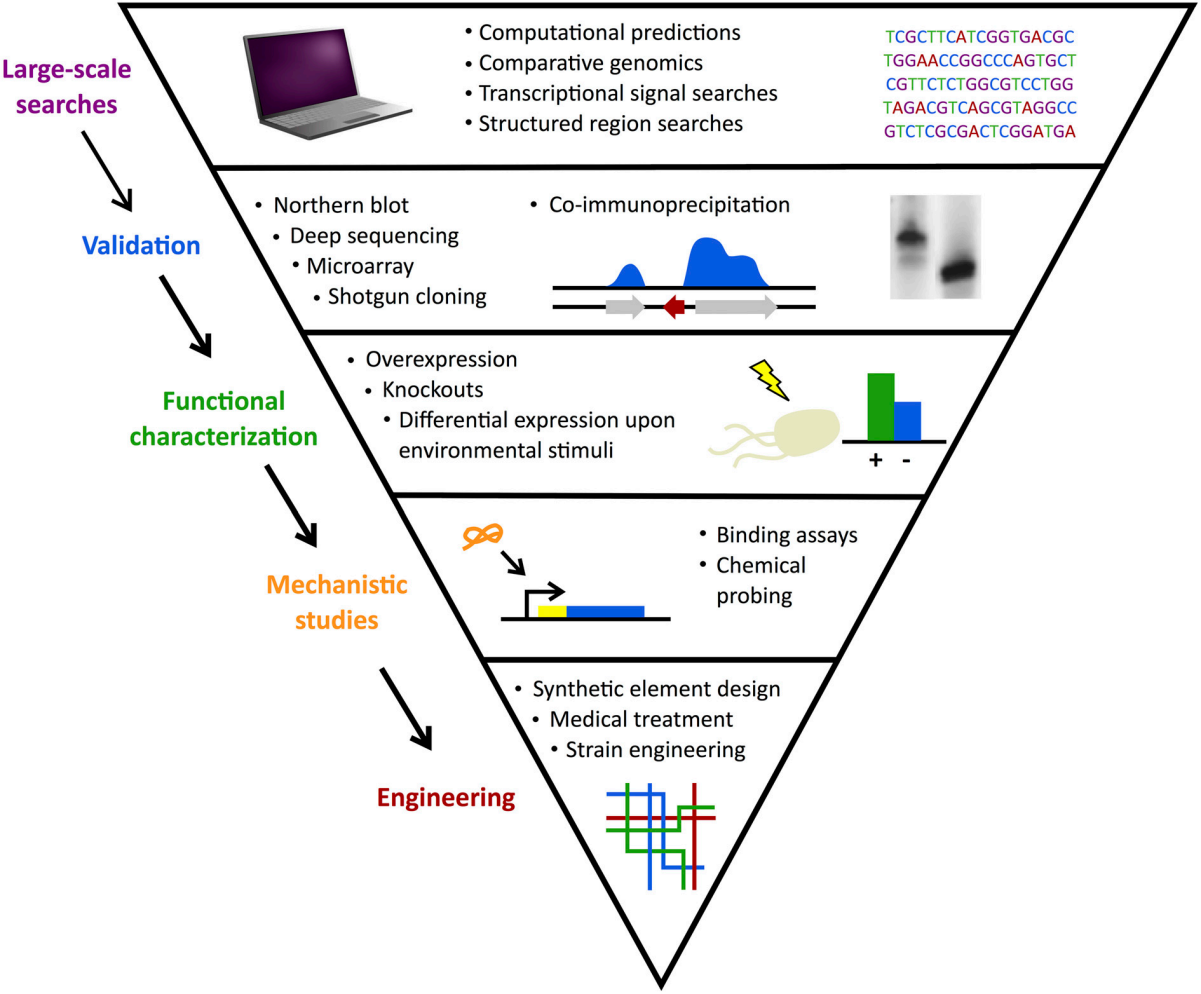
**sRNA search and characterization**. Discovery of sRNAs often begins with large-scale computational searches followed by experimental validation. Functional characterization of confirmed candidates identifies their gene or protein targets and mechanistic studies elucidate their methods of action. Finally, sRNAs can be used in engineering efforts to develop useful applications from synthetic elements to medical treatments.

With improved mechanistic understanding, there is enormous interest in inhibiting and/or mimicking expression of natural sRNA actions to achieve desired changes in targeted genes (Na et al., 2013; Vazquez-Anderson and Contreras, 2013). Figure 2 summarizes the large-scale sRNA searches, validation experiments, mechanistic and functional studies, and ultimately, targeting efforts. In parallel with such progress in model organisms, investigations emerged to follow this path in more unique classes of bacteria.

### sRNAs in pathogens pursued for medical advantages

The search of sRNAs has sparked special interest in the context of microbial pathogens. The emergence of studies of sRNAs in pathogenesis has been supported by the increasing availability of non-model microbial genomes as well as by the uncovered roles of sRNAs in environmental stress responses. For instance, the increasingly well-annotated collection of sRNAs in *E. coli* has facilitated discovery of sRNAs in other organisms by computational homology searches and other bioinformatic tools (Lu et al., 2011). Combining deep sequencing and transposon mutagenesis, a recent study identified 89 sRNAs in *Streptococcus pneumonia* and presented evidence of their importance in pathogenesis (Mann et al., 2012). Several investigations in non-model organisms have capitalized on the presence of Hfq in some pathogens to co-immunoprecipite bound RNA targets, followed by deep sequencing. In a model pathogen, *Salmonella enterica* serovar Typhimurium, this approach has revealed 100–200 sRNAs associated with Hfq and suggested that Hfq may regulate expression of more than one-fifth of all mRNAs (Sittka et al., 2008; Chao et al., 2012; Kröger et al., 2012). Although Hfq-dependent mechanisms have not yet been as prevalently detected in gram-positive bacteria (Romby and Charpentier, 2009), combined computational and experimental approaches have led to successful sRNA searches and validation in a variety of gram-positive pathogens that include *Staphylococcus aureus, Listeria monocytogenes*, and *S. pneumonia* (Papenfort and Vogel, 2010).

The sRNAs discovered in pathogens thus far exert diverse functions that include regulation of transcription factors, virulence genes, quorum sensing, and outer membrane dynamics in response to a variety of environmental inputs like temperature, pH, metabolite, oxidative, and anaerobic stresses. A few examples include the following: in *Helicobacter pylori*, a bacterial pathogen that colonizes the human stomach, a *cis*-encoded 5'*ure*B-sRNA enhances truncation of gastric acid acclimation operon *ureAB* at neutral pH, but releases its control in acidic environments to allow survival (Wen et al., 2012). Multiple *trans*-acting Qrr sRNAs in *Vibrio cholerae* collaborate with Hfq to regulate quorum sensing and biofilm formation, important for host transmission (Zhao et al., 2013). In *Shigella flexneri*, sRNAs CsrB and CsrC bind to protein CsrA to regulate carbon metabolism, attachment, and invasion in pathogenesis (Gore and Payne, 2010). Riboswitches may regulate 2% of genes in gram-positive pathogens such as *S. aureus* and *L. monocytogenes* in response to metabolites and pH (Caldelari et al., 2013). An RNA thermometer in *L. monocytogenes* inhibits translation of *prfA*, which encodes a key transcription factor for virulence (Johansson et al., 2002). Recent reviews provide more extensive catalogs of sRNA function and mechanisms in microbial pathogens (Gripenland et al., 2010; Papenfort and Vogel, 2010; Caldelari et al., 2013) as well as species-specific discussions for *L. monocytogenes* (Mellin and Cossart, 2012), *Pseudomonas aeruginosa* (Sonnleitner et al., 2012), *S. aureus* (Romilly et al., 2012; Tomasini et al., 2014), *S. Typhimurium* (Hébrard et al., 2012), and *V. cholerae* (Bardill and Hammer, 2012).

With increasing resistance of bacteria to traditional antimicrobials, sRNAs represent new medical targets to disarm pathogens. Riboswitches in particular are being pursued as antibiotic targets due to remarkable specificity and high affinity for their ligands (Lünse et al., 2014). At this point, one compound targeting the guanine riboswitch of *S. aureus* has been shown to successfully reduce bacterial concentrations in animals after infection (Ster et al., 2013). Also in *S. aureus*, sRNA SprX has been shown to regulate glycopeptide antibiotic resistance through antisense repression of sporulation protein SpoVG (Eyraud et al., 2014). This recent literature confirms the promise of sRNAs as new territory for controlling gene expression in pathogenesis.

## sRNAs in mycobacteria

Between 2003 and 2006, 30 new mycobacterial species were discovered, bringing the known total to 120 (Tortoli, 2006). These discoveries have resulted in significant investment toward understanding sigma factors and other regulatory proteins as potential keys to crippling virulence exerted by many of the mycobacterial pathogens (Rodrigue et al., 2006). Some common species that have been particularly studied are *M. tuberculosis, M. leprae*, and *M. ulcerans* due to their common pathogenesis in humans (Remus et al., 2003). *M. bovis* BCG is widely used as a vaccine against *M. tuberculosis* and is genetically similar, making it also a major focus of study (Skeiky and Sadoff, 2006). *M. smegmatis* serves as a model mycobacterial species due to ease of genetic manipulation, fast growth, and non-pathogenic nature (Shiloh and DiGiuseppe Champion, 2010).

### Early computational searches for sRNAs in mycobacteria

The growing interest in mycobacteria gene regulation has paralleled the launching of bioinformatics databases such as Rfam, a collection of non-coding RNA families that allows classification of new regulatory RNAs based on sequence and structure homology in over 200 complete genomes (Griffiths-Jones, 2005). One of the earliest mycobacterial genomes included in the Rfam database was *M. tuberculosis* CDC1551. This led to the prediction of three initial sRNAs in mycobacteria.

Although important for setting the stage to identify (and validate) the expression of more sRNAs in mycobacteria, the pure homology-based approach of Rfam limited early studies by having to rely on previously identified pools of sRNAs. A second type of bioinformatics search, sRNAPredict2, which predicted sRNAs by co-localization of genetic features common to sRNA-encoded regions, was also applied to the prediction of sRNAs in mycobacteria. This algorithm identified 56 new sRNAs in *M. tuberculosis* CDC1551, a much higher number that did not correspond to the three previously annotated by Rfam (Livny et al., 2006). Although these early computational efforts lacked consistency, most importantly, they provided evidence that multiple genomic regions could encode sRNAs in mycobacteria.

**Figure 3:**
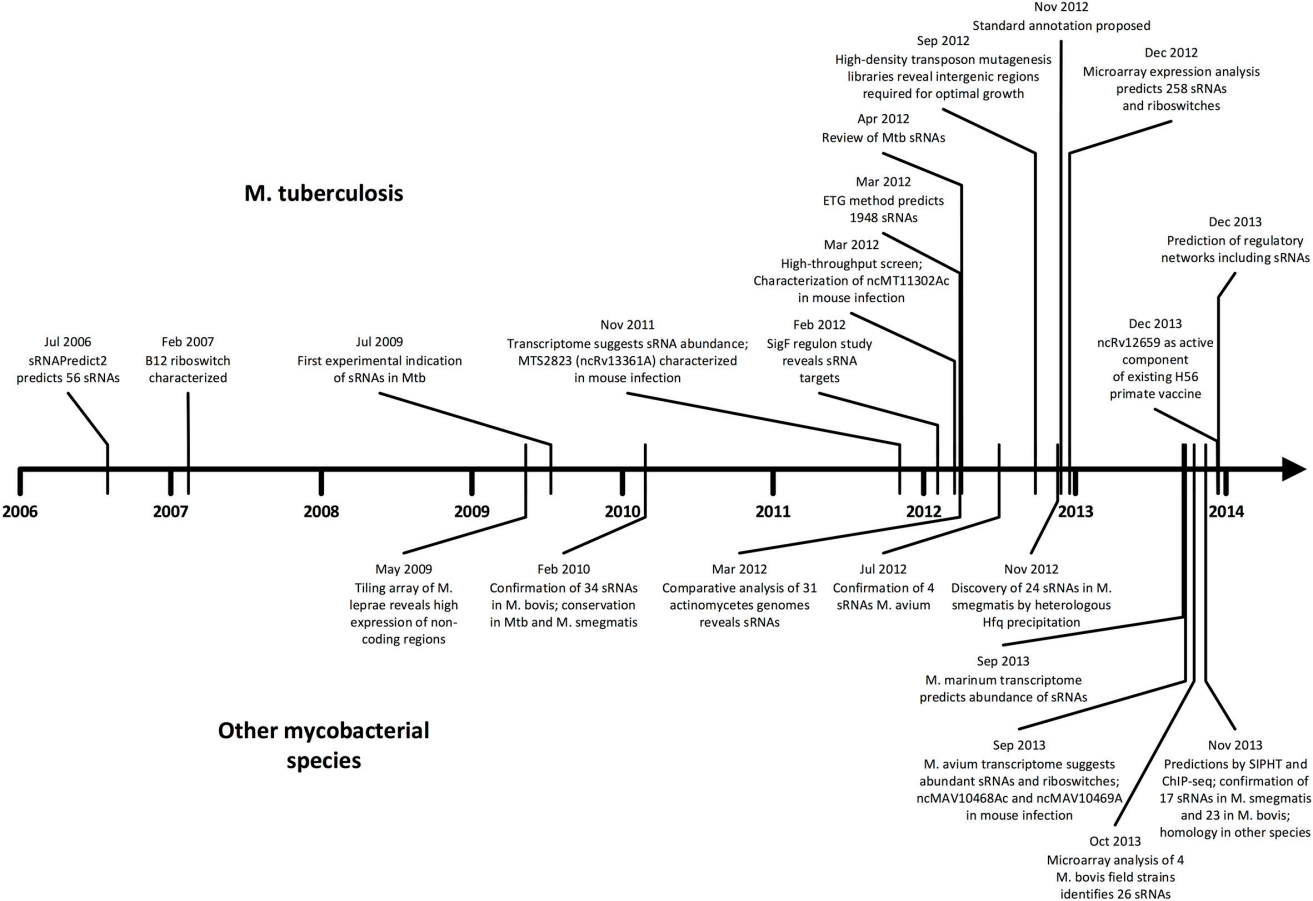
**Timeline of sRNA developments in mycobacteria**. Progress in *M. tuberculosis* (upper) and other mycobacterial species (lower) are shown in parallel in chronological order. The surge of studies in the last few years suggests momentum toward further discovery, mechanistic studies, and medical applications.

As we unfold major themes of the sRNA story in mycobacteria, refer to Figure 3 for the chronological alignment of these events. Also, note that Supplementary Table S1 lists all confirmed sRNAs in mycobacterial species to date along with characteristic data such as genomic location, differential expression, and known homology between species. Amazingly, more than half of the *M. tuberculosis* H37Rv sRNAs have been confirmed since the most recent sRNA review of this species (Arnvig and Young, 2012). Unconfirmed sRNA predictions are not included in Supplementary Table S1, but are quantified in Figure 1, a view of sRNA discovery by phylogeny within mycobacterial species, and listed in Supplementary Table S2. We use a universal sRNA nomenclature reported in Lamichhane et al. (2013) throughout this review to allow convenient cross-reference among the sRNAs being discussed.

### Early experimental indication of non-coding RNA regulators

One of the first experimental indications of the presence of responsive regulatory RNA elements in mycobacteria was obtained fortuitously by studies that aimed to understand the role of vitamin B_12_ in the growth inhibition of *M. tuberculosis* CD1551. In the context of this work, a B_12_ riboswitch (ncMT2183Ac) was found to regulate transcription of *metE*, a gene encoding methionine synthase (Warner et al., 2007). As previously suggested for other bacteria, this exposed potential for using antibiotics and/or small molecules to target riboswitch gatekeepers of essential genes expressed during pathogenesis. Only a couple of years after this study, tiling array analysis of *M. leprae* Thai-53 infected rats revealed high expression of 68 non-coding regions throughout the genome, representing 32.5% of the total relative expression profile (Akama et al., 2009). Although this data could have been interpreted as simply transcriptional degradation under these host conditions, earlier data in other mycobacterial species encouraged the alternative: that an abundance of sRNAs was expressed in this strain of *M. leprae*.

To date, riboswitch motifs remain largely uncharacterized in *M. tuberculosis* and further analysis of existing datasets could lead to confirmation of these important regulators. For example, two Mbox riboswitches (also called Ykok leaders) are predicted in *M. tuberculosis*: one located in the 5′ UTR of a hypothetical protein induced during Mg-starvation and the other in the 5′ UTR of a predicted magnesium transporter (Arnvig and Young, 2012). It has been hypothesized that these regulatory elements act similarly to their *Bacillus subtilis* homologs by inhibiting transcription of the downstream gene when bound to Mg^2+^.

### Confirmation of the first sRNAs in mycobacteria

The first complete experimental confirmation of sRNAs in mycobacteria was published in 2009, revealing five *trans*-encoded and four *cis*-encoded sRNAs in *M. tuberculosis* H37Rv (Arnvig and Young, 2009). These sRNAs were discovered by screening cDNA libraries of low molecular weight RNAs (20–75 nucleotides) in exponential and stationary growth phases. All reported sRNAs (see Supplementary Table S1) were confirmed by northern blotting analysis and their transcriptional ends were mapped by 5′ and 3′ RACE (Arnvig and Young, 2009). The functional significance of these sRNAs was reported in the context of oxidative stress, where *M. tuberculosis* H37Rv cells were cultured to exponential phase and then H_2_O_2_ was added to 10 mM for 1 h. Under these specific stress conditions, sRNAs B55 (ncRv10609AA), F6 (ncRv10243A), and ASpks (ncRv2048A) showed differential expression of 2-fold or greater (relative to their unstressed state). F6 was similarly expressed under pH stress, in which exponentially growing cells were suspended in acidified medium of pH 5.0 and allowed to grow for another 24 h. These conditions mimic the changing macrophage environment during active infection from pH ~6.2 to ~5.0 and during the accumulation of free fatty acids from the host (Vandal et al., 2009).

Evidence of the functional role of F6 (later reported as Mcr14) indirectly resulted from studies of the SigF regulon in *M. tuberculosis* H37Rv, where this sigma factor was shown to regulate its transcription (Hartkoorn et al., 2012). Furthermore, slow cell growth of H37Rv had been shown both upon F6/Mcr14 overexpression (under strong *rrnB* promoter of *M. smegmatis*) and upon induction of SigF (presumably due to its ability to up-regulate F6). It has also been suggested that some SigF binding sites are not associated with mRNA transcripts and are likely to bind to sRNAs not yet annotated.

Interestingly, none of the first experimentally confirmed sRNAs were predicted computationally. This discrepancy can be rationalized by the computational challenge presented by the uniqueness of the promoters and terminators in *M. tuberculosis*. Furthermore, the genomes of mycobacterial species are much higher in GC content (~65%) relative to other model bacteria (e.g., *E. coli*, GC content of ~50%) where earlier computational sRNA searches had been successful (DiChiara et al., 2010). For these reasons, one question of even more importance in mycobacterial species has been: How many computationally predicted sRNAs represent false positives? We have reported mixed results with predictions made by SIPHT and WU-BLAST (DiChiara et al., 2010; Tsai et al., 2013; Cho et al., 2014).

### A rising number of newly identified sRNAs

The year following the first report of sRNAs in *M. tuberculosis*, a new approach coupled low-molecular cloning and computational methods in *M. bovis* BCG, *M. tuberculosis* H37Rv, and *M. smegmatis* MC^2^155 (DiChiara et al., 2010). The cloning in these model strains began with construction and screening of cDNA libraries from both log and stationary phases, yielding 116 sRNA candidates. After elimination of rRNA, tRNA, and other annotated elements, 60 candidates were selected and tested in *M. bovis* by northern blotting analysis, leading to 19 confirmed sRNAs (listed in Supplementary Table S1). In parallel, the computational approach began with predictions by the SIPHT program, a high-throughput progeny of sRNAPredict2, which identified 144 sRNA candidates (Livny et al., 2008). The 67 that showed partial conservation in other mycobacterial species were tested by northern blotting in *M. bovis* and 21 additional sRNAs were confirmed (listed in Supplementary Table S1). Three sRNAs were confirmed by both cloning and computation methods, bringing the total to 37. Mcr6 (ncBCG3782Ac), Mcr14/Mpr13 (ncBCG10281A), and Mpr19 (ncBCG0526A) had been identified by previous studies in *M. tuberculosis* H37Rv as F6 (ncRv10243A), Mpr19 (ncRv13660Ac), and B11 (ncRv13660Ac), respectively. A unique approach taken by this study was to check for homologs of confirmed *M. bovis* sRNAs by using the same probes in northern blotting analysis in additional species. With this method, 20 new sRNAs were confirmed in *M. tuberculosis* H37Rv and 15 in *M. smegmatis* MC^2^155. The higher number identified and verified in *M. tuberculosis* (relative to *M. smegmatis*) is not surprising given the shorter evolutionary distance between *M. bovis* and *M. tuberculosis* relative to the distance between *M. bovis* and *M. smegmatis*, as shown in Figure 1 (Devulder, 2005). Only three *M. bovis* sRNAs were identified by both cloning and computational methods and only three *M. tuberculosis* H37Rv sRNAs had been previously discovered, conveying the importance of combining various approaches.

A follow-up study (Tsai et al., 2013) used the same SIPHT predictions in *M. bovis* BCG and *M. smegmatis* to test the remaining candidates. As part of that work, 23 additional novel sRNAs were confirmed by northern blotting analysis in *M. bovis* BCG and 17 in *M. smegmatis* MC^2^155 (listed in Supplementary Table S1). With *M. smegmatis* as the primary species of interest, this approach revealed nine homologs in *M. bovis* and four in *M. tuberculosis* (listed in Supplementary Table S1). By analysis of existing ChIP-seq data from *M. tuberculosis* (Galagan et al., 2012), at least four sRNA 5′ ends were matched with otherwise uncharacterized transcription factors. From this series of studies, a stronger connection between pathogenesis and differential expression of sRNAs in mycobacteria emerged. A powerful example was the differential expression of Mcr11, an sRNA between two cAMP metabolism genes, in *M. bovis* (ncBCG1323Ac) and *M. tuberculosis* (ncRv11264Ac) under host-like conditions, such as low pH (5.5) and hypoxia (1.3% O_2_ + 5% CO_2_). Perhaps most importantly, this study proposed the use of conservation analysis across multiple mycobacterial species to begin posing questions related to the potential pathogenic vs. housekeeping functions of the newly uncovered sRNAs. New questions were raised for future studies that included: Which sRNAs are uniquely conserved among the pathogenic species? Do conserved sRNAs by sequence perform the same function across phylogeny? This study also was one of the first to adhere to the recommended annotation (also being used in this review).

### Implications of a missing Hfq homolog to the search and characterization of sRNAs

A challenge in mycobacterial sRNA studies has been the lack of identification of an Hfq homolog, preventing Hfq co-immunoprecipitation approaches for sRNA discovery. The lack of Hfq has also raised questions about alternative chaperones or inherent stability granted by C-rich stretches observed in the rising number of confirmed sRNAs. It is important to note that in 2012, a review of non-coding RNAs specific to *M. tuberculosis* (Arnvig and Young, 2012) posed the notion that hypothetical protein Rv2367 of unknown function could serve as a potential RNA chaperone alternative to Hfq based on its homology to YbeY, a protein in *Sinorhizobium meliloti* shown to perform certain Hfq-like functions (Pandey et al., 2011). Rv2367, however, has significantly lower expression than Hfq in *E. coli*, strengthening the argument that C-rich stretches of mycobacterial sRNAs may provide enough stability to the sequences without the need of a functionally-equivalent chaperone.

It is also worth noting that a novel method has exploited the conserved sRNA-binding ability of *E. coli* Hfq by expressing it in *M. smegmatis* and using co-immunoprecipitation and deep sequencing to identify sRNAs (Li et al., 2012). The approach found 12 *trans*-encoded and 12 *cis*-encoded sRNAs confirmed by northern blotting analysis and mapped by 5' RACE (listed in Supplementary Table S1). Interestingly, five of the *cis*-encoded sRNAs are immediately upstream of known transposases. Some of the identified *M. smegmatis* sRNAs also show homology to regions in other mycobacteria, especially in non-pathogenic species known for rapid growth, suggesting that these sRNAs could be involved in enhancing growth efficiency. All of them showed differential expression between exponential and stationary phases. Homologs to previously identified sRNAs C8 (ncRv13722Ac) and B11 (ncRv13660Ac) in *M. tuberculosis* H37Rv (Arnvig and Young, 2009) were also identified in *M. smegmatis* (ncMSMEG16286A and ncMSMEG6172Ac, respectively). Consistent with previous studies, multiple sizes of individual sRNAs were also observed here, raising questions about how sRNAs are processed and the potential role of sigma factors in mycobacteria. Although, sRNA regulation of transposition has been studied in *E. coli*, mechanisms in mycobacteria remain largely obscure.

### An explosion in sRNA identification guided by high-throughput sequencing

Transcriptome profiling of *M. tuberculosis* H37Rv revealed high-density reads in intergenic regions (other than from those encoding rRNAs and tRNAs), representing a potential abundance of sRNAs (Arnvig et al., 2011). In fact, 28% of the total transcriptome represented intergenic reads, consistent with other organisms. The most highly expressed sRNA detected in exponential phase was MTS2823 (ncRv13661A), which also increased 10-fold in stationary phase. Overexpression of MTS2823 up-regulated two genes (Rv2035, a potential activator of HspG, and Rv3229c, a fatty acyl desaturase) and down-regulated a large set of about 300 genes, including a methyl citrate synthase that is reduced by 15-fold. It was suggested that MTS2823 may have functional homology with 6S RNA, but the mechanism remains unclear. MTS1338 (ncRv1734A) was also induced in stationary phase, depending at least partially on the DosRS hypoxia-responsive regulator system. In the same study, significant accumulation of sRNAs was observed in infected lungs of mice, especially MTS2823, MTS0997 (ncRv11264Ac), and MTS1338, suggesting potential involvement in pathogenesis. Later work showed that *M. tuberculosis* H37Rv sRNAs MTS0479 (ncRv10609AA) and MTS1338 are not present in *M. avium* (Ignatov et al., 2013). Since *M. avium* is conditionally pathogenic and *M. tuberculosis* H37Rv is highly pathogenic, these two sRNAs could be key players in pathogenesis. These studies marked an important step in the successful application of high-throughput genome-wide sequencing technology for sRNA identification as well as in establishing direct medical relevance to pathogenesis by use of mouse models.

The next genome-wide search in *M. tuberculosis* examined strain CDC1551 (Pelly et al., 2012). Twelve sRNA candidates were identified; four of the intergenic candidates were novel sRNAs and the other eight all contained sequences within the region between genes MT1302 and MT1303, which showed homology to a single sRNA identified in *M. bovis* BCG. This abundant sRNA was labeled ncrMT1302 (ncMT1130Ac) and examined further under nitric oxide (250 μM diethylenetriamine nitric oxide adduct), low pH (5.5), and limited nutrition (PBS + 0.05% Tween 80) stresses for 6 h during exponential phase. Expression levels of ncrMT1302 were reduced in all cases, most dramatically at low pH with a 7-fold reduction. To detect antibiotic responses, total RNA was exposed to the minimal inhibitory concentrations (MIC) of isoniazid or rifampicin for 3 h before ncrMT1302 expression was observed by northern blot analysis. Isoniazid showed no significant effect, but rifampicin caused reduction in ncrMT1302 expression. MT1302 encodes an adenylyl cyclase that converts ATP to cAMP and MT1303 encodes a hypothetical protein that is transcribed in the presence of cAMP in low-oxygen conditions. In a strain lacking the cAMP-producing gene MT1302, ncrMT1302 was less abundant and no longer differentially expressed under stress. This sRNA also contains a potential binding site for transcription factor Cmr (cAMP and macrophage regulator) and was confirmed in the lungs of mice during infection.

A new method for sRNA identification involved building an effective target genome (ETG) and then combining transcriptome data with comparative genomics (Pellin et al., 2012). The target genome in this case included only intergenic regions, thus coding regions were removed before the dataset was filtered further by RNA-seq reads and conservation between genomes. Applied to *M. tuberculosis* H37Rv, this approach generated a list of 1948 candidate sRNAs, which included six of the nine identified by Arnvig and Young (Arnvig and Young, 2009). This method also predicted 17 of the 37 found in *M. bovis* BCG by DiChiara et al. (2010). All candidates identified were compared with the Rfam database and many showed homology with known families. Most notably, Rfam matches included SAM-IV, TPP family, and Ykok leader riboswitches.

In addition to large scale transcriptomic studies, a comparative analysis of 31 genomes of mycobacteria and related actinomycetes by sequence and gene expression allowed identification of 50 predicted non-coding RNAs (McGuire et al., 2012). This set of genomes included 8 strains of the *M. tuberculosis* complex (*M. tuberculosis* and *M. bovis*) and 11 other mycobacteria including *M. leprae, M. ulcerans, M. marinum, M. avium*, and *M. smegmatis*. Four more novel sRNAs were confirmed by northern blotting analysis in *M. tuberculosis* H37Rv (listed in Supplementary Table S1) with homology in *M. smegmatis* MC^2^155 (listed in Supplementary Table S2). This massive dataset highlighted the evolutionary importance of lipid metabolism and its regulation among these species in regards to pathogenesis. Figure 1 illustrates the presence of sRNAs across mycobacterial phylogeny.

### Increased studies beyond *M. tuberculosis, M. bovis*, and *M. smegmatis*

By cloning and homology search, four intergenic sRNAs have been directly detected in *M. avium* subsp. avium TMC724 (listed in Supplementary Table S1) (Ignatov et al., 2012). Because the genome of this strain is not available, the sRNAs were mapped by RACE to the *M. avium hominissuis* 104 genome. A follow-up study of *M. avium* TMC724 predicted 86 *cis*-encoded sRNAs, 10 *trans*-encoded sRNAs, and five riboswitches, which are listed in Supplementary Table S2 (Ignatov et al., 2013). The predicted riboswitches included three Ykok leaders (Mg^2+^ sensing) and one SAM-IV (S-adenosylmethionine sensing); both types had been previously spotted in *M. tuberculosis* H37Rv. The *trans*-encoded sRNAs showed conservation with *M. tuberculosis* and *M. ulcerans*. Of six intergenic sRNAs, only igMAV_1034-1035 (ncMAV11034Bc) had no homolog in *M. tuberculosis*. The two mostly highly expressed sRNAs in this study, igMAV_0468-0469 (ncMAV10468Ac) and igMAV_0469-0470 (ncMAV10469A), were tested in mice genetically susceptible and genetically resistant to *M. avium* infection. The resistant mice showed much lower expression of these sRNAs in lung tissue relative to non-resistant mice. However, the *M. tuberculosis* homolog to igMAV 0468-0469, sRNA MTS2823 (ncRv13661A), was shown to accumulate to high levels in the lungs of mice resistant to tuberculosis, potentially indicating a difference between *M. avium* and *M. tuberculosis* in the mouse infection model.

In addition to increased studies in *M. avium*, transcriptome analysis has been recently applied to the search of regulatory sRNAs in *M. marinum* during exponential and stationary phases. In these studies, it was observed that read-rich antisense and intergenic regions accounted for 23% of the total transcriptome in exponential phase and 40% in early stationary phase (Wang et al., 2013). A very highly expressed sRNA candidate, MMAR_5556, is homologous to MTS2823 (ncRv13661A), previously shown as the most abundant sRNA in *M. tuberculosis* H37Rv. Three sRNAs predicted were matched with Rfam as a TPP family riboswitch, an ALIL pseudoknot, and a 6C RNA.

### Efforts to unify rapidly increasing knowledge in the field

As focus begins to shift to the functional characterization of mycobacterial sRNAs, the need to unify all research efforts in cross-species discovery and characterization has become more prevalent. A challenge highlighted by increased sRNA discovery studies across mycobacterial species was the lack of universal sRNA nomenclature across the emerging literature. For instance, F6, Mcr14, Mcr13, and MTS0194 represent the same sRNA (ncRv10243A). To address this issue, an official recommendation for systematic annotation of non-coding RNA in mycobacteria was recently published (Lamichhane et al., 2013). The suggested format includes species designation and genomic location. For example, an sRNA identified within the open reading frame (ORF) of gene Rv1234 in *M. tuberculosis* H37Rv would be labeled “ncRv1234” if on the plus strand or “ncRv1234c” if on the minus strand. If the sRNA were located completely between ORFs, it would carry the number of the upstream ORF and a “1” would be added in front to indicate an intergenic region, such as “ncRv11234” or “ncRv11234c.” Finally, the direction of the sRNA should be indicated by “A” if in the plus strand orientation or “B” if in the minus strand orientation, such as “ncRv1234A” or “ncRv11234B.” In this way, the sRNA names would give useful information and be consistent between studies. We have adhered to this nomenclature in this review, where we have taken the challenge to begin consolidating all mycobacterial sRNAs.

### From discovery to functional characterization

As seen above, *cis*-regulatory element functions are suggested by their locations relative to known ORFs. Although not greatly exploited in mycobacterial species, bioinformatics tools such as TargetRNA have been used in *Salmonella* and *Listeria* pathogens to predict sRNA interactions *in silico* (Tjaden, 2008; Wurtzel et al., 2012; Yu and Schneiders, 2012). Still, experimental methods are necessary to confirm these suggested interactions. Efforts to elucidate sRNA functions have traditionally relied on microarray for transcriptome analysis under various stress conditions or under sRNA overexpression or deletion. Even with the development of high-density tiling arrays, this technology is becoming obsolete in favor of increasingly affordable high-throughput methods.

The sRNA candidates identified with the ETG transcriptome and comparative genome approach in *M. tuberculosis* H37Rv (Pellin et al., 2012) have been examined by microarray to confirm expression (Miotto et al., 2012). Of the 1373 predicted in exponential phase, 258 were confirmed by microarray including 22 intergenic, 84 in 5′ or 3′ UTRs (including potential riboswitches), and 152 antisense sRNAs. Twenty of 23 candidates tested were confirmed by northern blot analysis and mapped by 5′ RACE (listed in Supplementary Table S1). A computational analysis of pathway regulation showed that membrane-bound proteins were especially likely subjects of antisense regulation. Additionally, about 100 sRNAs < 50 nucleotides were detected, but the functions of these micro-sized RNAs are unknown.

In an effort to define genomic regions required for growth in *M. tuberculosis* H37Rv, two 100,000-clone libraries were generated by high-density transposon mutagenesis (Zhang et al., 2012). Transposon-mapping probes were developed and amplified to allow deep sequencing of their neighboring genomic regions, mapping insertion sites. Sliding window analysis was used to scan the genome for insertion site counts in order to find underrepresented regions, likely to be essential in function. The search identified 25 intergenic regions as essential for growth including tmRNA, the RNA component of RNaseP, and 19 regions with unknown function. One limitation of this study was that the smallest search window for intergenic regions was 250 basepairs, preventing detection of shorter sRNAs. This study showed consistency with a previous microarray approach (Sassetti et al., 2003), but provided much higher resolution.

Perhaps the most currently favored high-throughput alternative to microarray is ChIP-seq, which combines traditional chromatin immunoprecipitation with deep sequencing to map *in vivo* DNA-protein interactions across the genome. Advantages of ChIP-seq over microarray include single-nucleotide resolution (compared to 30–100 bp resolution for microarray), low cost, and small amount of required DNA (10–50 ng for ChIP-seq compared to >1 μg for microarray) (Park, 2009).

By combining ChIP-seq and microarray data, sRNA regulation networks were predicted for *M. tuberculosis* H37Rv and are consistent with previously known sRNA functions (Peterson, 2013). The context likelihood of readiness (CLR) algorithm was used to infer the networks and false positives were filtered out by comparison to overexpression microarray data. As a proof-of-concept, *M. tuberculosis* was grown under hypoxia stress over 14 days and examined by microarray, revealing 58 *trans*-acting sRNAs significantly induced or repressed, and the sRNA hypoxia regulatory network was successfully generated. These networks can be visualized in a spider web fashion to clearly show direct and indirect connections. Sigma factor SigH was predicted to regulate sRNA ncRv13596A, linking hypoxia and cholesterol metabolism, and DosR was predicted to regulate ncRv1102A and ncRv1734A, linking hypoxia also to changing phthiocerol dimycocerosates. SigF and ncRv10243c were associated with the cell wall and plasma membrane, consistent with *E. coli* homologs with a known mechanism.

In other organisms, ultraviolet crosslinking and immunoprecipitation (CLIP) has been used to detect RNA-protein binding sites *in vivo* with deep sequencing. Photoactivatable ribonucleoside-enhanced CLIP (PAR-CLIP) allows single cross-linked nucleotide resolution, but requires cells to incorporate photoactivatable nucleoside analogs like 4-thiouridine or 6-thioguanosine (König et al., 2012). CLIP-seq provides strand-specific data unlike ChIP-seq (Wang et al., 2014). At this point, CLIP has been most used to determine alternative splicing mechanisms in mammalian systems (König et al., 2014). Mycobacterial sRNA studies may pursue PAR-CLIP or other CLIP variations to obtain strand-specific binding insight in the future. Other technologies are available for characterization of discovered sRNA interactions including reporter gene fusions (*gfp, lacZ, luc*), *in vitro* RNA-RNA footprinting, and *in vitro* toeprinting (Podkaminski et al., 2014). In particular, toeprinting has been successful in characterizing sRNAs that negatively regulate their targets. With these techniques, mycobacterial studies will benefit from precedents set in widely studied organisms.

### sRNA characterization for medical applications

Recent studies have employed high-throughput sRNA characterization methods to analyze medical problems. Remarkably, in response to an epidemic of bovine tuberculosis in Great Britain, four field strains of *M. bovis* were processed by sequencing, microarray, transcriptome analysis, RACE, and recombinant DNA technology to observe effects of synonymous point mutations (sSNPs) (Golby et al., 2013). Unexpectedly, some of these sSNPs of coding strands resulted in expression of antisense transcripts on the opposite strand, challenging the definition of “silent mutation” and suggesting the potential of sRNAs in these loci. A high-density tiled microarray was designed to examine changes in expression of non-coding RNAs across the genomes. Transcripts were considered confirmed sRNAs only if detected by two or more overlapping probes. In this way, 26 sRNAs were found (listed in Supplementary Table S1). Seven intergenic transcripts were located within the direct repeat (DR) locus, a suggested CRISPR locus. One antisense sRNA as_Mb1618c (ncMb1618Ac) overlaps with a predicted secretory lipase gene was only present in strain 1121/01. Similarly, as_Mb1914c (ncMb1914Ac) and as_echA21 (ncMb3803Ac) were only expressed in strain 2451/01, encoded antisense to a short chain dehydrogenase and an enoyl-CoA hydratase, respectively. These sRNAs unique to individual strains may be evolutionary adaptations to different environments.

The H56 vaccine for primate *M. tuberculosis* includes Rv2660c as an antigen, which overlaps with sRNA ncRv12659 (Houghton et al., 2013). The Rv2660c locus was originally selected as an antigen due to its enhanced transcription during *M. tuberculosis* H37Rv starvation, but by RNA-seq and northern blotting, the sRNA was shown to be the true cause of this increased expression. Because ncRv12659 accumulates to high levels during infection, it can serve as a potential biomarker. Still, the mechanism by which ncRv12659 leads to this increased transcription is unknown. Interestingly, only the 5′ portion of ncRv12659 was detected during infection, raising questions about sRNA processing. Truncation could be the result of premature termination or degradation of an unstable 3′ portion.

## The path forward

The surge of sRNA studies in mycobacteria in the last 3 years favors continued momentum toward medical applications. Still, many species have yet to be studied and a large number of sRNAs remain uncharacterized. High-throughput experimental methods would aid in bridging the gap between the ever-growing pool of predicted sRNA candidates and the set of confirmed molecules with known functions. Work remains to be done computationally and experimentally to map the complex sRNA control networks of pathogens. Future work could employ ChIP-seq to map gene regulatory networks and truly begin addressing the need to understand the functional role of these sRNAs in mycobacterial species. Likewise, increased phenotypic data for individual sRNA knockout or overexpression strains could enhance efforts to map sRNA regulatory networks.

Despite the limited number of functionally characterized sRNAs in mycobacteria, the door is already open for exploiting known sRNA functions in pathogens to gain medical advantages. The development of antibiotics to cripple sRNA-enabled virulence could be a powerful, new approach, especially as microbes show resistance to conventional compounds. In particular, abundant and uniquely expressed sRNAs during infection could serve as useful biomarkers or as therapeutic targets.

### Conflict of interest statement

The authors declare that the research was conducted in the absence of any commercial or financial relationships that could be construed as a potential conflict of interest.
